# Traditional Chinese medicine in diabetes management: a comprehensive review of mechanisms and therapeutic potential

**DOI:** 10.3389/fendo.2026.1709404

**Published:** 2026-03-03

**Authors:** Yuzhe Fan, Shengjie Gong, Xu Wang

**Affiliations:** 1 The First Clinical Medical College, Heilongjiang University of Chinese Medicine, Harbin, China; 2 Department of Pharmacology, College of Basic Medicine, Heilongjiang University of Chinese Medicine, Harbin, Heilongjiang, China

**Keywords:** diabetes mellitus, diabetic complications, elderly, herbal therapy, traditional Chinese medicine

## Abstract

**Background:**

Diabetes mellitus is a chronic metabolic disorder frequently associated with severe complications. Traditional Chinese Medicine (TCM) offers potential therapeutic benefits through multi-targeted mechanisms.

**Objective:**

This study explores the pharmacological actions, clinical applications, and therapeutic potential of TCM herbs, extracts, and prescriptions in managing diabetes-related complications.

**Methods:**

A comprehensive review of studies from multiple databases was conducted, focusing on experimental evidence, signaling pathways, and clinical trials evaluating TCM interventions for diabetes and its complications.

**Results:**

TCM demonstrates significant potential in modulating insulin secretion, improving glucose metabolism, and attenuating oxidative stress. Specific formulations and bioactive compounds regulate pathways including PI3K/Akt, Nrf2/HO-1, and VEGF, contributing to better glycemic control and reduced complications such as diabetic retinopathy, cardiomyopathy, osteoporosis, and peripheral neuropathy. Several randomized controlled trials report enhanced clinical outcomes when TCM formulations are used alone or in combination with conventional therapy.

**Conclusion:**

TCM offers a promising complementary strategy for diabetes management and its associated complications. Further large-scale clinical trials are needed to validate efficacy and ensure safety.

## 1 Introduction

Type 2 diabetes mellitus (T2DM) is a chronic metabolic condition that has become a major global health concern, with profound effects on health outcomes and quality of life. The rising prevalence of T2DM has increased the challenges faced by healthcare providers, requiring comprehensive strategies to achieve stringent glycemic control, prevent complications, and improve patient well-being (1). Despite the use of traditional oral hypoglycemic drugs, insulin therapy, and lifestyle interventions, many patients continue to experience suboptimal outcomes, highlighting the need for alternative treatment approaches (2). This has prompted the medical field to explore innovative therapeutic options that go beyond conventional methods.

Traditional Chinese Medicine (TCM), with its rich cultural heritage and centuries of clinical application, offers a holistic model for managing diabetes. Historically, the relationship between TCM and diabetes dates back to the Han Dynasty, where the famous physician Zhang Zhongjing doccessive heat in the lung and stomach, and “Shenqi pills” for kidney qi deficiency syndrome (3).

The TCM framework addresses much more than glucose control, emphasizing individualized, patient-focused approaches aimed at preventing complications, improving quality of life, minimizing adverse drug effects, and delivering flexible yet precise blood glucose management (4). A notable strength of TCM lies in its ability to enhance pancreatic islet activity and regulate neuroendocrine pathways mechanistic characteristics that continue to attract the interest of modern diabetes scholars (5, 6). This review aims to provide a comprehensive overview of the role of TCM in diabetes care, categorizing its therapeutic mechanisms into key areas: glycemic control, cardiovascular protection, and neuroprotection. In addition to examining the evidence supporting these mechanisms, this review also assesses the potential of integrating TCM with conventional therapies to enhance clinical outcomes for diabetes patients.

## 2 Methods

A comprehensive literature search was performed using publicly accessible databases, including PubMed, ScienceDirect, and Google Scholar. The search strategy employed Medical Subject Headings (MeSH) in combination with additional keywords such as “Traditional Chinese Medicine,” “TCM,” “TCM herbs,” “diabetes mellitus,” “diabetic complication,” “diabetic retinopathy,” “diabetic neuropathy,” “diabetic nephropathy,” and “diabetic cardiomyopathy.” These MeSH terms were integrated using Boolean operators to identify publications relevant to the research objectives. Studies were included if they focused on the pharmacological effects of TCM in managing diabetes mellitus or its complications, were published in peer-reviewed journals in English or other relevant languages, and involved clinical trials, preclinical studies, or meta-analyses. Studies were excluded if they were unrelated to diabetes or TCM, or had incomplete data or low methodological quality, such as non-randomized studies or small sample sizes. The scope of the search included review articles, meta-analyses, and original research studies. This study is presented as a narrative review summarizing recent advances in the application of TCM for diabetes and its complications, with a particular focus on therapeutic mechanisms.

### 2.1 Generation of supplementary tables

The supplementary tables, including **Spplementary Table File Tables 1–3** were generated by extracting and synthesizing key information from the studies selected according to the inclusion/exclusion criteria. The herbs listed in these tables were identified through a systematic review of primary research studies, meta-analyses, and other relevant sources. The herbs were categorized based on their therapeutic mechanisms, such as metabolic regulation, antioxidant activity, and inflammatory pathways modulation, as reported in the literature. Information from multiple studies was compiled to highlight the most frequently used herbs and their therapeutic effects in the context of diabetes treatment and management.

### 2.2 Age-related pharmacokinetic and pharmacodynamic changes in elderly diabetic patients

As individuals age, significant changes in pharmacokinetics (PK) and pharmacodynamics (PD) may influence the effectiveness and safety of medications, including TCM. Elderly patients often experience altered drug metabolism due to decreased liver function, slower renal clearance, and reduced intestinal absorption. These age-related physiological changes necessitate careful consideration when applying TCM treatments in older populations.

In addition to PK changes, pharmacodynamic alterations in older adults, such as reduced receptor sensitivity and impaired enzyme function, can modify the response to therapeutic agents. Common comorbidities such as hypertension, cardiovascular diseases, and kidney dysfunction further complicate the pharmacological management of diabetes in this population. Moreover, polypharmacy, the concurrent use of multiple drugs, increases the risk of drug interactions, which may alter the efficacy of TCM interventions. Thus, personalized approaches to TCM dosing and monitoring are crucial for ensuring safety and maximizing therapeutic benefits in elderly diabetic patients.

#### 2.1.1 TCM mechanisms in glucose regulation

##### 2.1.1.1 Traditional Chinese medicines, classical formulas, and mechanistic insights into blood glucose regulation in T2DM

**Figure 1:**
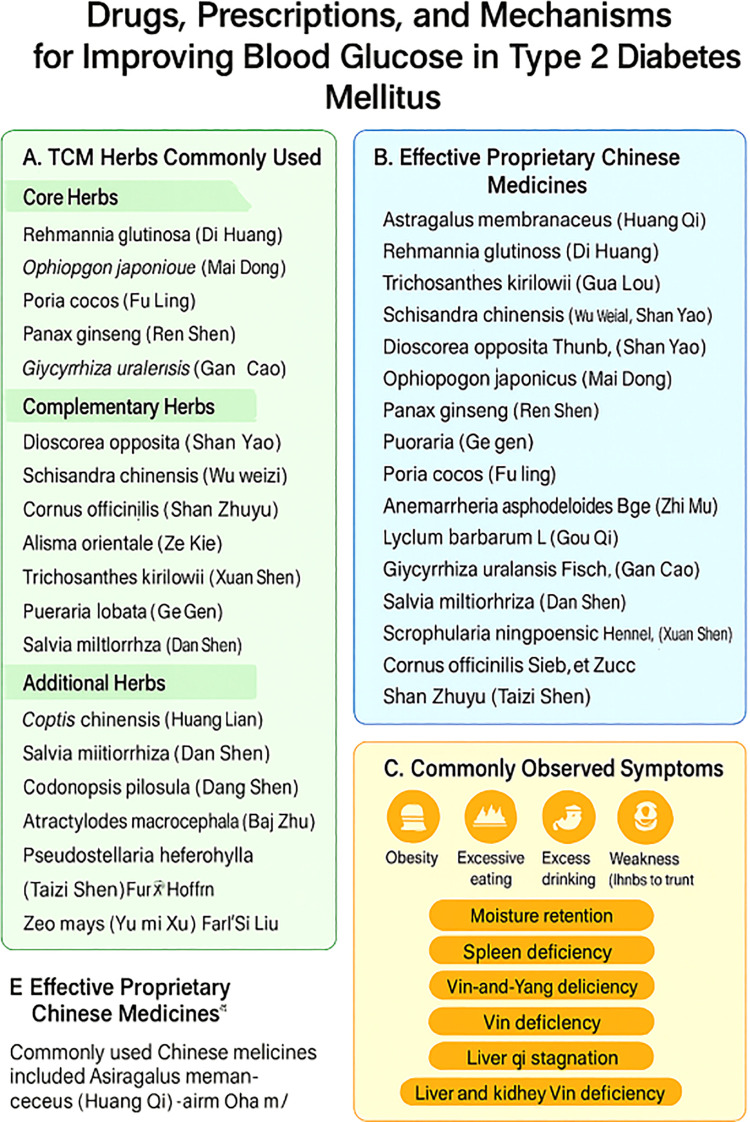
Drugs, prescriptions, and mechanisms for improving blood glucose in Type 2 Diabetes Mellitus through Traditional Chinese Medicine (TCM). **(A)** Commonly used TCM herbs categorized into core herbs, complementary herbs, and additional herbs frequently applied in treatment. **(B)** List of effective proprietary Chinese medicines derived from commonly used herbs for blood glucose regulation. **(C)** Overview of commonly observed symptoms in patients with Type 2 Diabetes Mellitus, including obesity, excessive eating and drinking, weakness, and associated syndromes such as moisture retention, spleen deficiency, Yin-Yang imbalance, liver qi stagnation, and liver-kidney Yin deficiency. **(E)** Highlights the proprietary Chinese medicines most commonly used in clinical practice for improving glycemic control.

A recent meta-analysis examined the most commonly reported TCMs used in diabetes care, historically referred to as “dissipating thirst syndrome” (7). This review highlighted several herbal agents, which can be categorized under key therapeutic mechanisms: Metabolic Regulation, Antioxidant Activity, and Inflammatory Pathways Modulation. These herbs include Rehmannia glutinosa (Di Huang), Ophiopogon japonicus (Mai Dong), Poria cocos (Fu Ling), Panax ginseng (Ren Shen), Astragalus membranaceus (Huang Qi), and Glycyrrhiza uralensis (Gan Cao).A separate review on proprietary Chinese medicines reported frequent use of herbs such as Rehmannia glutinosa (Di Huang), Trichosanthes kirilowii (Gua Lou), Schisandra chinensis (Wu Wei Zi), and Dioscorea opposita (Shan Yao), which fall under similar therapeutic categories. These herbs contribute to the regulation of blood glucose through insulin secretion, glucose uptake, and oxidative stress modulation. More details can be found in **Supplementary Table 2**. Another meta-analysis on prevalent clinical symptoms in diabetes outlined obesity, polyphagia, polydipsia, polyuria, fatigue, and persistent thirst as the most common presentations of T2DM (8).These symptoms are often linked to metabolic imbalances like moisture retention, spleen deficiency, Yin-Yang deficiency, and liver qi stagnation. These syndromes, identified in TCM, guide the formulation of herbal prescriptions tailored to manage the diverse symptoms of T2DM (see **Figure 1**).Classical TCM prescriptions, refined over centuries, continue to play a key role in managing diabetes. For instance, herbal combinations such as Coptis chinensis, Rheum palmatum (Da Huang), Bupleurum chinense (Chai Hu), and Astragalus mongholicus (Huang Qi) remain crucial for diabetes care. These formulas contain bioactive compounds like flavonoids, terpenoids, alkaloids, curcumin, and cinnamaldehyde, which work synergistically to lower blood glucose levels and improve metabolic functions (7, 8).

**Figure 2:**
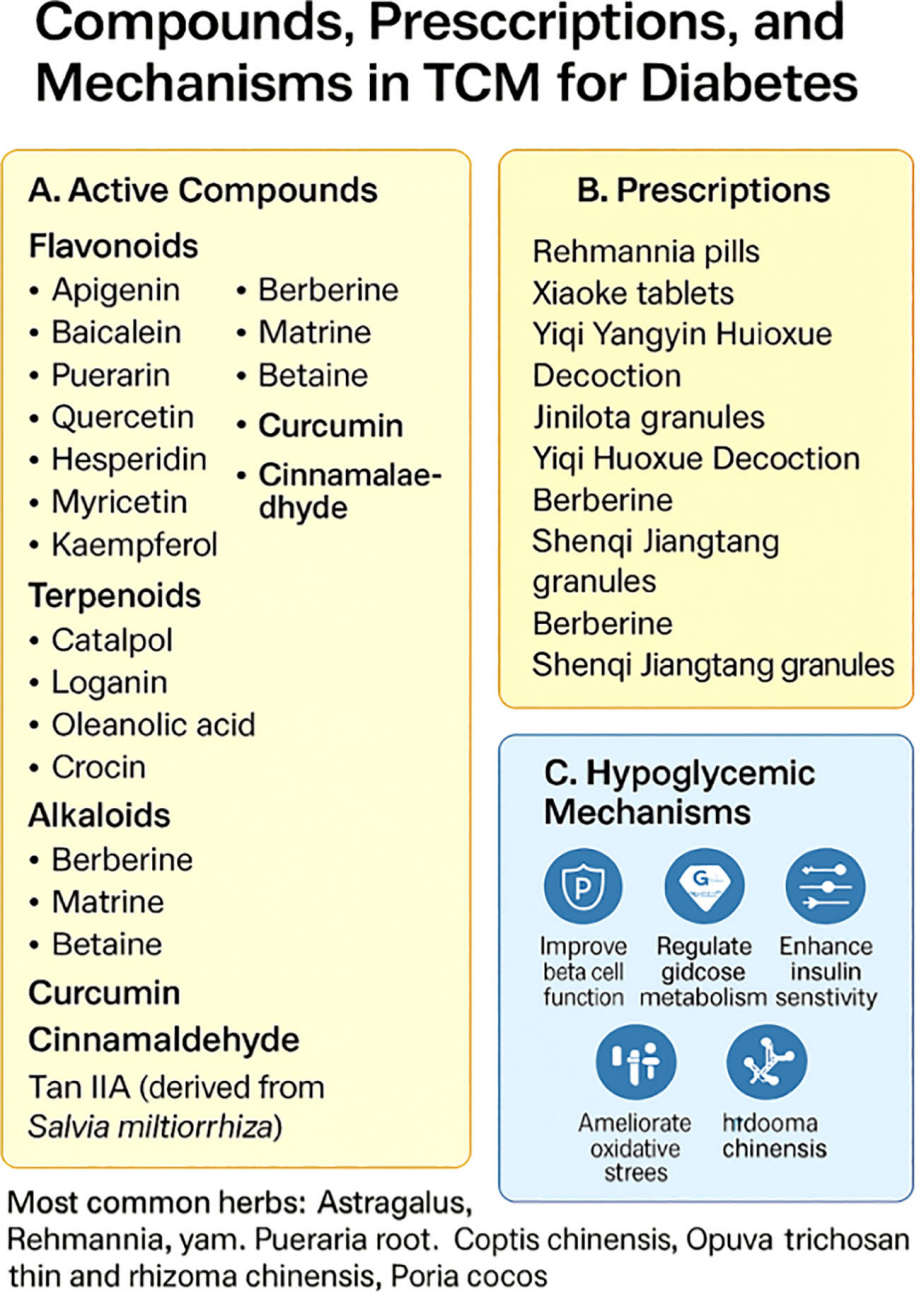
Drugs, prescriptions, and mechanisms for improving blood glucose in Type 2 Diabetes Mellitus through Traditional Chinese Medicine (TCM). **(A)** List of commonly used TCM herbs, categorized into core herbs, complementary herbs, and additional herbs applied in the treatment of hyperglycemia. **(B)** Overview of effective proprietary Chinese medicines formulated from these herbs and their relevance in glycemic control. **(C)** Summary of commonly observed clinical symptoms in patients with Type 2 Diabetes Mellitus, including obesity, excessive eating and drinking, and weakness, along with syndromic patterns such as moisture retention, spleen deficiency, Yin-Yang imbalance, liver qi stagnation, and liver-kidney Yin deficiency. **(E)** Highlights the proprietary Chinese medicines frequently adopted in clinical practice for regulating blood glucose and improving overall metabolic function.

The glucose-lowering potential of TCM agents operates via multiple biochemical pathways, impacting both fasting and postprandial glycemic control. Recent advances in metabolomics are enhancing the identification of active components and molecular targets involved in glucose homeostasis. These findings open new possibilities for minimizing diabetes-related complications, suggesting that TCM’s role in glucose regulation may extend beyond traditional treatments (see **Figure 2**).

##### 2.1.1.2 Mechanisms of action of TCM in T2DM

TCM encompasses a wide range of herbal formulations that exert diverse effects on glucose regulation, insulin sensitivity, and pancreatic β-cell function. These effects can be grouped into three broad thematic categories: Metabolic Regulation, Antioxidant Activity, and Inflammatory Pathways Modulation.

###### 2.1.1.2.1 Metabolic regulation

TCM herbs influence insulin secretion, improve glucose metabolism, and enhance insulin sensitivity. This is achieved through modulation of key signaling pathways such as PI3K/Akt, which governs glucose uptake, glycogen synthesis, and β-cell function. Herbs like Panax ginseng and Astragalus membranaceus play significant roles in regulating these processes.

###### 2.1.1.2.2 Antioxidant activity

Oxidative stress is a major contributor to the progression of T2DM. TCM herbs such as Rehmannia glutinosa and Lycium barbarum are known for their potent antioxidant effects. These herbs activate the Nrf2/HO-1 pathway, which reduces oxidative damage to pancreatic β-cells, thereby improving insulin sensitivity and preventing complications like diabetic retinopathy, nephropathy, and neuropathy.

###### 2.1.1.2.3 Inflammatory pathways modulation

Chronic inflammation is a critical factor in insulin resistance and the progression of diabetes. TCM herbs such as Salvia miltiorrhiza and Poria cocos modulate inflammation by regulating cytokine production and inhibiting NF-κB activation, which leads to reduced inflammation and improved insulin sensitivity.

**Figure 3:**
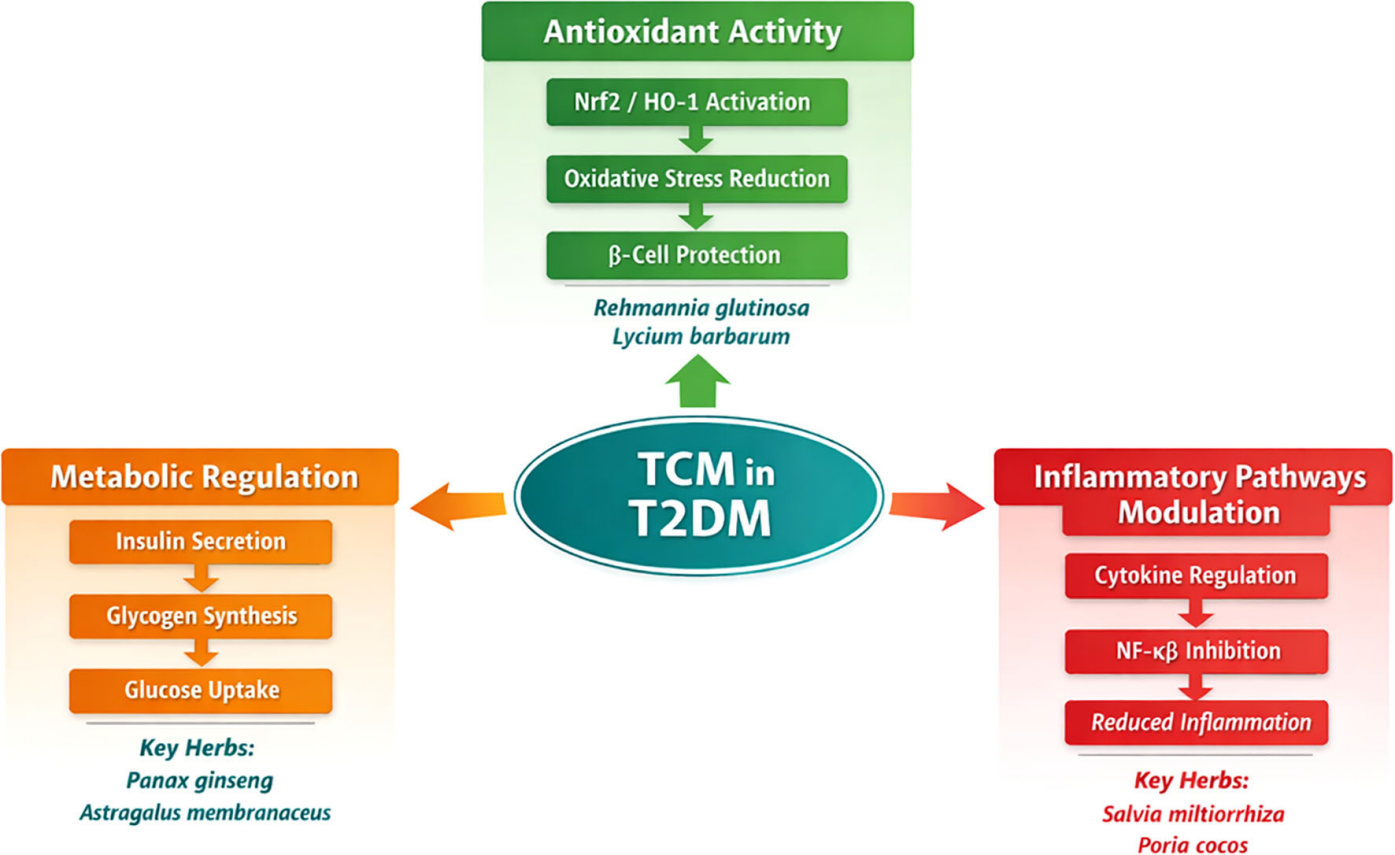
this figure illustrates the mechanisms through which Traditional Chinese Medicine (TCM) influences the management of Type 2 Diabetes Mellitus (T2DM). It categorizes the mechanisms into Metabolic Regulation (orange), Antioxidant Activity (green), and Inflammatory Pathways Modulation (red). These categories represent key processes such as insulin secretion, glucose metabolism, oxidative stress reduction, and inflammation modulation. The diagram also highlights the herbs associated with each mechanism: Panax ginseng and Astragalus membranaceus for metabolic regulation, Rehmannia glutinosa and Lycium barbarum for antioxidant activity, and Salvia miltiorrhiza and Poria cocos for inflammatory pathways modulation.

These therapeutic effects result from molecular modulation and broader metabolic pathway regulation, offering complementary benefits to conventional diabetes management approaches **Supplementary Table 3**; **Figure 3**.

#### 2.1.2 TCM approaches for managing metabolic risks and diabetic complications

##### 2.1.2.1 Stimulation of insulin secretion

Several TCM herbs have been reported to promote insulin release, primarily through the modulation of the PI3K/AKT signaling pathway, which plays a key role in glucose uptake, glycogen synthesis, and gluconeogenesis. Key herbs such as Coptis chinensis, Pueraria, Astragalus, and Rehmannia have demonstrated significant insulin-stimulating effects. These herbs contain bioactive compounds, including flavonoids, polyphenols, alkaloids, terpenoids, and saponins, which contribute to enhancing insulin secretion and improving glucose metabolism (7, 9, 10).

##### 2.1.2.2 Pancreatic β-cell protection and α-glucosidase inhibition

Formulations like the Shenqi compound (SQC) have been shown to lower blood glucose, enhance insulin sensitivity, and protect pancreatic β-cells by alleviating oxidative stress and inflammation, while reducing β-cell apoptosis and senescence (7). Puerarin also plays a role in maintaining stable glycemic control by stimulating β-cell formation and elevating GLP-1 levels. In addition, several TCM herbs, including Cornus officinale, Galla chinensis (Wu Bei Zi), and Morus alba (Sang Ye), have demonstrated significant inhibitory effects on α-glucosidase activity, which helps delay carbohydrate digestion and absorption, thereby reducing postprandial glucose surges (7).

##### 2.1.2.3 Management of obesity and related metabolic risks

Obesity and metabolic syndrome, significant contributors to T2DM, are addressed in TCM through a comprehensive approach that integrates herbal remedies, lifestyle adjustments, acupuncture, Tuina massage, Tai Chi, and dietary recommendations. TCM botanicals are noted for their safety, tolerability, and sustainable weight management benefits. Key agents such as ginseng, bitter melon, and cinnamon (Gui Pi) have demonstrated significant effects on lipid and glucose metabolism, energy regulation, and insulin sensitivity. Other herbs, including Rhizoma coptidis, Semen cassiae (Jue Ming Zi), Fructus crataegi (Shan Zha), Radix puerariae, and Radix astragali, also contribute to improving metabolic function (11).

Metabolic-associated fatty liver disease (MASLD/MAFLD) is closely linked to insulin resistance and T2DM. In addition to managing weight and metabolic risks, TCM strategies focusing on improving liver metabolism show promise, with findings suggesting a potential overlap with incretin-based therapeutic mechanisms. TCM interventions have been shown to enhance glucose, lipid, and oxidative stress profiles while favorably modulating gut microbiota (12–15). These interventions influence gut–brain axis hormones, immune responses, and inflammatory signaling, ultimately supporting both hepatic and systemic metabolic function.

##### 2.1.24 Modulation of glucose metabolic factors and related pathways

Murrayae Folium et Cacumen extracts, rich in flavonoids, improve metabolic markers by optimizing glucose and lipid metabolism, reducing oxidative stress, and mitigating inflammation (16). Another key herb, Dioscorea opposita (Huai Yam), enhances glucose absorption and insulin sensitivity while providing antioxidative and anti-inflammatory benefits. Similarly, Astragalus, a widely used TCM herb, improves both metabolic and cardiovascular health (17).

Panax notoginsenoside R1 (NG-R1), derived from Panax notoginseng, activates key pathways such as PI3K/AKT, Wnt/β-catenin, and NRF2-HO-1, which reduce inflammation, apoptosis, and oxidative damage, and help protect podocytes and retinal tissue from hyperglycemia-related injury (18).

##### 2.1.2.5 Insights from modern metabolomics

Recent metabolomics studies have highlighted the bioactive constituents and mechanistic pathways of classical TCM prescriptions, such as Ge-Gen-Jiao-Tai-Wan (GGJTW) and Huanglian Decoction, which are associated with key processes like bile acid production, branched-chain amino acid metabolism, and anti-inflammatory actions (19, 20). Bioactive compounds including berberine, baicalin, and wogonin have been shown to activate AMPK, regulate GLUT transporter expression, and modulate gut microbiota composition, further supporting their therapeutic potential. Specifically, Ge-Gen-Qin-Lian Decoction (GGQLD) improves intestinal barrier function and reduces inflammation, while Astragalus membranaceus and Lian-Ge granules target insulin signaling and lipid metabolism through multi-pathway modulation (21–23). These interventions also influence gut microbiota, enriching butyrate-producing bacteria and reducing lipopolysaccharide-producing species, which contribute to enhanced insulin sensitivity and metabolic regulation (24).

#### 2.1.3 TCM Interventions for diabetic complications

##### 2.1.3.1 TCM-based approaches for cardiovascular protection and diabetic osteoporosis

The degeneration of vascular structures and their cellular components is a notable feature in older individuals with diabetes (25, 26). Several TCM herbal preparations, including ginseng, notoginseng, and Ligusticum chuanxiong, have been shown to protect endothelial cells from damage caused by hyperglycemia and hyperlipidemia. These herbs operate through mechanisms such as stimulation of autophagy, enhancement of mitochondrial membrane potential, reduction of DNA damage from reactive oxygen species (ROS), and improvement of cardiac aging and vascular calcification. Additionally, vascular aging may be delayed through AMPK/mTOR pathway inhibition and modulation of TXNIP, NF-κB signaling, and the NLRP3 inflammasome, with concurrent activation of Nrf2-dependent antioxidant defenses.In diabetic osteoporosis, TCM herbs such as Epimedium brevicornum, Phellodendron chinense, and Anemarrhena asphodeloides target bone remodeling and reduce osteoporotic changes. Epimedium exerts effects through the Bax/Bcl-2 signaling cascade (27, 28), while Phellodendron chinense arabinoxylans act on the AGE/RAGE signaling axis (29–31). *Both herbs, along with Phellodendri chinensis cortex, influence key pathways like NLRP3, ASC, caspase, GSDMD, and Nrf2-Keap1, which promote bone health and reduce inflammation. Other herbs like Rehmannia and Ligustrum lucidum support calcium homeostasis and enhance parathyroid hormone (PTH) secretion (*32, 33). A meta-analysis of TCM formulas for osteoporosis in elderly diabetic patients identified Epimedium, Angelica sinensis, Rehmannia, and Astragalus as key therapeutic ingredients, with Qianggu Capsules, Jintian Capsules, and Tangmaikang Granules showing clinical efficacy in alleviating discomfort, improving bone density, and reducing blood glucose levels (34). Flavonoid-rich extracts from Drynaria roosii Nakai (Drynaria rhizoma) have been linked to improved bone remodeling via activation of the BMP2/Smad signaling cascade.

##### 2.1.3.2 Preventing muscle loss and preserving insulin sensitivity

Both *Astragalus* and yam have demonstrated protective effects against diabetes-related muscle wasting by modulating mitochondrial function through the Rab5a/mTOR signaling axis (35). In animal studies, *Radix vasculus* and *Radix phellodendri* have been shown to counteract muscle atrophy via regulation of the Akt/mTOR/FoxO3 pathway (36).

##### 2.1.3.3 Optimizing gut microbiota composition

Phytochemicals from *Scutellaria baicalensis*, such as baicalin, can elevate levels of short-chain fatty acids (SCFAs) generated by intestinal microorganisms (37).

Alkaloids from mulberry branches and polyphenols from Magnolia officinalis, including magnolol, help modulate gut microbiota by promoting a balanced distribution of beneficial flora. Additionally, Qingke, rich in protein and β-glucan, supports glycemic regulation and microbial equilibrium. These herbs, along with resveratrol and Poria cocos, exert anti-inflammatory effects and restore intestinal barrier integrity by inhibiting the LPS-binding protein–MCP-1–CD14–TLR4 cascade and suppressing LPS–TLR4 signaling, which alleviates TSDM symptoms.

Gegen Qinlian Decoction has been shown to exhibit hypoglycemic effects, reduce LPS and inflammatory markers, and induce favorable shifts in the microbiome. Furthermore, modulation of the gut microbiota is associated with improved hormone secretion and gastrointestinal motility. For instance, Shenlingbaizhu powder has been observed to reduce motilin levels while increasing somatostatin in obese T2DM patients with spleen deficiency and moisture stagnation.

##### 2.1.3.4 TCM in managing diabetic complications

Traditional Chinese Medicine provides distinctive benefits in addressing diabetic complications offering vascular protection, mitigating vascular deterioration, preventing kidney injury (38), and influencing autophagy-related pathways (39). Literature reviews have outlined TCM bioactives such as saponins (40), luteolin (41), and kaempferol (42) that contribute to these therapeutic effects.

##### 2.1.3.5 TCM in diabetic nephropathy

A meta-analysis of 37 randomized controlled trials assessed the efficacy of several TCM formulations, including Yishenhuashi Granules, Huangkui Capsules, and Jinlida Granules, which are commonly used in DN treatment (34). A systematic review identified critical inflammatory pathways such as TLR, NLRP3, Nrf2, AMPK, and AGE/RAGE as key therapeutic targets in DN management (43). TCM interventions typically focus on promoting fluid circulation, nourishing blood and Yin, detoxifying, and reducing swelling (43, 44). Representative formulas such as Zishen-wan, which regulates PI3K/Akt and p38 MAPK pathways, and Xiexin Decoction, which downregulates NF-κB and improves mesangial proliferation and basement membrane thickening, demonstrate efficacy in regulating these pathways (45, 46).

Jiangtang Decoction and Danzhi Jiangtang Capsule target PI3K/Akt and JAK2–STAT1/STAT3–SOCS3 signaling, enhancing insulin sensitivity and reducing AGE accumulation and COX-2 expression (47, 48). Other formulations, such as Sanzi Guben Granule, increase Nrf2 activation and modulate GSH and catalase levels to mitigate oxidative stress and inflammation (49, 50).

Formulas targeting TGF-β/Smad and TGF-β1/SGK1 pathways, including Naoxinton Capsule, Huangkqi Decoction, and Tongxinluo, help reduce extracellular matrix deposition, inhibit EMT, and protect podocytes (51, 52). Additional prescriptions like Zhenwu Decoction, Yishen Capsules, and Bushen Huoxue Decoction preserve podocyte integrity through RAS inhibition, autophagy enhancement, and reversal of podocyte damage markers (53, 54). Qigui Tangshen Granules and Jowiseungki also promote gut health by regulating gut microbiota and reducing inflammation, further supporting kidney function (55–57).

##### 2.1.3.6 Treating DN by TCM herbs or extracts

A meta-analysis of 18 randomized controlled trials on senile diabetic nephropathy (DN) identified Astragalus, yam, and Cornus officinalis as the most frequently used TCM herbs for DN treatment. The herbal extract Fucodian (from Hakim Shieu capsules) has been shown to reduce inflammatory markers and delay renal function decline via the AMPK–ULK1 signaling pathway (57). Additionally, Ligustrum chuanxiong extract attenuates oxidative stress and inflammation through the Nrf2 and NF-κB pathways (58),while chronic hyperglycemia activates NADPH oxidase (NOx), contributing to kidney injury (59).

Key TCM formulations such as Bombax ceiba ethanol extract and mangiferin help suppress ROS production and oxidative stress by inhibiting NOX4, and Nepeta angustifolia has been shown to inhibit peroxidase-induced apoptosis by modulating mitochondrial potential (60). Nelumbo nucifera leaf extract activates the Akt pathway, suppressing oxidative stress and apoptosis, and protecting renal tissues (61, 62).

Further treatments targeting TGF-β/Smad and NLRP3 inflammasome include Dioscorea zingiberensis phenolic extract, which modulates P2X7 receptor expression and improves insulin resistance and fibrosis (63–66). Additionally, Polygonum cuspidatum extract improves insulin resistance and nephritis while inhibiting TGFβ/Smad signaling and reducing oxidative stress (67, 68). Formulas like Epigallocatechin-3-gallate, Schisandra chinensis extract, and Panax notoginseng promote podocyte integrity and reduce inflammation via various mechanisms, including suppression of EMT and HIF1-α/VEGF pathways) (69, 70). Panax notoginseng suppresses HIF1-α and VEGF, upregulates nephrin, and ameliorates tubulointerstitial injury (70). Coptis chinensis promotes podocyte autophagy via AMPK activation and mTOR suppression (71).

Several TCM compounds, including Epigallocatechin-3-gallate, Schisandra chinensis extract, and Panax notoginseng, help preserve podocyte integrity by inhibiting epithelial–mesenchymal transition (EMT) and modulating HIF1-α/VEGF pathways. Additional compounds like Curcumin, Dihydroquercetin, and Berberine modulate AMPK, NF-κB, and NLRP3 inflammasome activity, producing significant antifibrotic and anti-inflammatory effects (72–75).

Ginsenoside Rg1, Icariin, and astragaloside IV (AS-IV) promote podocyte autophagy and reduce oxidative damage through AMPK–mTOR, NF-κB, and PDK1–Akt signaling pathways (76, 77). Other bioactive compounds like artesimisin, bergenin, naringenin, and quercetin target TGF-β/Smad and HIPPO pathways, modulating ECM turnover and reducing inflammation (78, 79).

Notable bioflavonoids, such as puerarin found in Nelumbon nucifera and Plantago asiatica, reduce fibrogenetic effects by modulating transcription factors, inflammation, oxidative stress, fibrosis, and promoting autophagy (80, 81). Catalpol, from Rehmannia, influences multiple pathways (NF-κB, SIRT3/AMPK, AMPK/SIRT1/NF-κB, TGF-β/Smad, RhoA/ROCK) and structural proteins (nephrin, podocin), supporting cytoskeletal stabilization and macrophage infiltration (82, 83).

Loganin, from Cornus officinalis, reduces mesangial proliferation, oxidative stress, and fibrosis by modulating CTGF, AGE–RAGE, and NLRP3/caspase-1 signaling (84, 85). Additional agents like swertiamarin, gentiopicroside, and oleuropein exert anti-inflammatory and antiapoptotic effects via MAPK, TGF-β/Smad, and NF-κB modulation (86–93). Morroniside (MOR) from Cornus officinalis lowers glucose, proteinuria, and urea nitrogen, restores podocyte function, and ameliorates lipid toxicity–induced renal damage through PGC-1α-mediated pathways (94, 95).

##### 2.1.3.7 TCM for diabetic retinopathy

A meta-analysis of elderly patients with diabetic retinopathy (DR) identified three clinical studies involving Danhuang Mingmu Decoction and Zhenwu Decoction (34). Among single prescriptions, Danhong Huayu Oral Liquid was shown to suppress inflammatory responses and oxidative stress while downregulating TNF-α expression (95). Similarly, Mingmu Decoction reduced proinflammatory gene levels within the TNF-α regulatory network (96), whereas Danggui Buxue Decoction significantly decreased retinal TNF-α expression in GK rats, mitigating high-glucose-induced endothelial cell migration and proliferation (97). Other classical prescriptions target distinct molecular pathways. Tonglu Tangjing may inhibit retinal MMP-2/9/13 activity through the MMP/ICRI axis (98), while kidney-tonifying and blood-activating formulas (Tonifying Kidney and Huoxue) attenuate oxidative damage in the ocular fundus of DR models (99). Jianxuanqingping prescription reduces retinal microvascular permeability by upregulating tight junction proteins, suppressing the AGE–RAGE axis, and reducing inflammation and apoptosis in endothelial cells (100). Xueshuantong and its core formulation, Fufang Xueshuantong, help protect against DR by modulating TGF-β/Smad2/3 oxidative stress pathways and reducing VEGF expression through a YAP-mediated mechanism (101, 102). Fushuning Capsules improve blood flow and inhibit fibrosis by suppressing VEGF, and VCAM-1 (103). Whereas Danshen dripping pills and Naoxintong Capsules prevent retinal atrophy and the formation of acellular capillaries in DR mice via inhibition of caspase-3, MMP-2/9, and the accumulation of carbohydrate-related macromolecules (104). The Huoxue Jiedu Recipe further demonstrates efficacy by reducing hyperglycemia-induced apoptosis, and promotes extracellular matrix stability by restoring the TIMP1–A2M–induced apoptotic pathway and balancing MMP-2/9 activity (105).

##### 2.1.3.8 Mechanistic insights into TCM-based therapies for diabetic retinopathy

Multiple studies have elucidated the pharmacological mechanisms by which TCM extracts exert therapeutic effects in diabetic retinopathy (DR) (106). For instance, Lycium barbarum polysaccharides (LBP) modulate the Rho/ROCK inflammatory signaling pathway, protect the blood–retinal barrier, increase Bcl-2, decrease Bax levels, and attenuate apoptosis (107). Likewise, ω-3 fatty acids in flaxseed oil alleviate DR-related retinal dysfunction by suppressing inflammation and upregulating GPR120 expression (108). Anti-inflammatory actions are also observed with several herbal extracts. The ethanol extract of Sannai inhibits ERK1/2 and NF-κB signaling to reduce retinal inflammation (109). Scutellaria increases claudin-1 and claudin-19 expression, counteracting TJ disruption caused by INF-4, PKC, and NF-κB, thereby improving vascular permeability (110). Moreover, Polygonum cuspidatum extract inhibits the HMGB1–RAGE–NF-κB pathway to suppress inflammation and prevent DR-associated increases in vascular permeability (111). Since ischemia–hypoxia-induced neovascularization is a hallmark of DR progression, herbal agents such as erianthrine inhibit capillary regeneration in the posterior pole region of DR rats (112), while Vaccinium myrtillus, Lonicera japonica Flos suppress angiogenesis by downregulating PKCβ2 and modulating VEGF expression (113). Dendrobium administration also inhibits VEGF/VEGFR2 and other proangiogenic mediators, including MMP-2/9, PDGF A/B, EGF, and IGF-1 (114), while Typhae pollen polysaccharides improve hemodynamic parameters by reducing hypoxic injury (115). Plantaginis semen extract reduces inflammation by downregulating NF-κB and lowering ICAM-1 and VCAM-1 expression (116). The Nrf2/HO-1 pathway is another major therapeutic target in DR. Crude saponins from Panax notoginseng and blueberry anthocyanins activate Nrf2/HO-1 to combat oxidative stress (117), while notoginsenoside R1 reduces retinal ganglion cell apoptosis by inhibiting the eIF2α/ATF4/CHOP mitochondrial stress pathway (117). Other natural compounds intervene in polyol and oxidative stress cascades. Litchi chinensis pericarp extracts, and phenolic compounds including and rographolide, inhibit aldose reductase (118), whereas ginsenoside Rb1 and Pterocarpus marsupium extracts activate the NAD–PARP–SIRT axis, enhancing antioxidant capacity (119). Ginsenoside Rg3 reduces VEGF and TNF-α levels (120), while Astragaloside IV and luteolin downregulate Nox4 expression (121). Astragaloside polysaccharides regulate miR-122 and miR-195 to protect mitochondrial function and reduce apoptosis (122). Berberine and notoginsenoside R1 further regulate PARP activity (123, 124), while berberine additionally activates AMPK (125). Hesperidin restores mitochondrial membrane potential (126), and puerarin suppresses iNOS to reduce oxidative damage in retinal pigment epithelial (RPE) cells (127). Isanhuo Jitia protects RPE cells from hypermethylglyoxal-induced oxidative stress (128) and calycosin modulates NLRP3 inflammasome activation (129). Meanwhile, multiple agents modulate inflammatory signaling cascades: Fangchinoline inhibits RAGE–NF-κB, curcumol attenuates ROS–Akt/mTOR (130), and gastronin, astragalus total saponins, and baicalin target the TLR4–NF-κB pathway (131). Ginkgo biloba extract reduces inflammation (132), while specnuezhenide delays angiogenesis by modulating HIF-1α/VEGF signaling (133). Chlorogenic acid downregulates Notch1 signaling, and Lycopus lucidus inhibits VEGF/VEGFR2-mediated angiogenesis by suppressing hypoxia-induced stress (134). Other agents, such as berberine, rutsin, and notoginsenoside R1, modulate angiogenesis through the AMPK pathway (135), whereas Astragalus polysaccharides regulate caspase activity and luteoloside increases Bcl-2 expression by modulating C/EBP signaling (117).

##### 2.1.3.9 TCM-based prescriptions and clinical evidence for diabetic peripheral neuropathy

Several clinical investigations have evaluated the efficacy of TCM prescriptions, herbal components, and extracts in the treatment of diabetic peripheral neuropathy (DPN), particularly in older adults. In one randomized controlled trial (RCT) involving 68 patients with DPN, berberine not only improved blood glucose and 24-hour urinary protein levels but also enhanced motor nerve conduction velocity (MNCV) and sensory nerve conduction velocity (SNCV). These neuroprotective effects were associated with modulation of the PI3K/Akt/Bcl-2, Nrf2/HO-1, and MAPK signaling pathways (34). Results from individual trials indicated that aloe did not significantly improve overall DPN symptoms, though it enhanced conduction velocities in the fibular and median nerves. Ciwujia, when combined with vitamins B1 and B12, demonstrated greater symptom improvement compared to vitamin therapy alone, while Erigeron showed superior symptom relief in alleviating DPN symptoms. Similarly, Ginkgo biloba combined with B vitamins did not show a significant advantage over vitamin B injection alone. In studies of TCM patent medicines, outcomes varied. Qiying granules showed no significant difference from cobamamide granules, while Fufang Danshen injection combined with mecobalamin improved symptoms more effectively than mecobalamin alone. Furong Tongqai capsules produced greater symptom relief and improved conduction velocity in both sensory and motor fibers of the common peroneal nerve compared with mecobalamin injection. Tangluoan capsules improved sensory and motor nerve conduction in the common peroneal and median nerves relative to placebo, but did not outperform adenosylcobalamin in overall symptom relief, although conduction velocity gains were superior. Conversely, Tongmai capsules failed to improve symptoms versus adenosylcobalamin, and Tongxiling capsules combined with mecobalamin offered no additional symptom improvement beyond mecobalamin monotherapy. Some formulations demonstrated better combined effects with vitamin therapy. For example, the combination of vitamins B1 and B12 was more effective than either treatment alone, and Xuefuzhuyu capsules with mecobalamin did not significantly increase benefits. Xuetaosan, which contains notoginseng saponins and flavonoids, in capsule form added to mecobalamin showed no significant improvement, though injectable formulations with vitamins B1 and B12 did enhance symptom relief. Buyang Huanwu Tang with mecobalamin yielded clear improvements in symptoms and common peroneal nerve conduction velocity, and a modified version of Buyang Huanwu decoction combined with vitamins B1, B6, and B12 achieved greater symptom improvement than vitamin therapy alone. Other formulations with positive results included Jianbi Yiqi Tongluo Tang, which improved symptoms more effectively than adenosylcobalamin; modified Danggui Sini Tang, which surpassed vitamin monotherapy; and improved Huangqi Guizhi Wuwu Tang with mecobalamin, which outperformed mecobalamin alone in one study, though another trial found no advantage over B1 and B6 therapy. The improved Sishen Jian showed notable improvement versus B1 and B12 therapy, while Tangmai Yin provided better symptom relief than mecobalamin, though nerve conduction benefits were mainly limited to distal sensory conduction velocity. Taoren Honghua Jian with mecobalamin significantly improved symptoms, as did Tongbi Tang, which was superior to both mecobalamin and vitamin B1. However, some formulas showed no superiority to conventional therapy.

##### 2.1.3.10 TCM-based interventions for diabetic cardiomyopathy

Recent clinical studies have investigated the efficacy of TCM in treating diabetic cardiomyopathy (DCM) among older patients. Findings indicate that the Yangyin Yiqi Huoxue Formula enhances cardiac performance in individuals with coexisting hyperglycemia and heart failure, potentially through the regulation of vascular endothelial growth factor (VEGF) signaling. Similarly, the Zhigancao Decoction has been shown to alleviate arrhythmias and improve autonomic nervous system stability, while concurrently reducing systemic inflammation (34).

##### 2.1.3.11 Diabetic peripheral vascular disease

TCM interventions have also been investigated in elderly patients with diabetic peripheral vascular disease. Three randomized controlled trials assessed modified Huangqi Guizhi Wuwu Decoction and Tangmai Tongluo Decoction in the treatment of lower extremity vascular lesions, reporting improved blood flow to the affected limbs. In addition, Yiqi Tongluo Qingre cream demonstrated efficacy in improving arteriosclerosis of the common carotid, popliteal, and dorsal foot arteries, while also enhancing lipid metabolism profiles.

Four studies on proprietary Chinese patent medicines included Naoxintong capsule, Yixinshu capsule, Yangxinshi tablet, and Shexiang Baoxin pill. Furthermore, four RCTs involving a total of 432 elderly patients evaluated extracts from notoginseng, and Ginkgo biloba demonstrating therapeutic potential for vascular health in this patient population (34).

##### 2.1.3.12 Antioxidant effect and mechanism of TCM

TCM interventions have also been investigated in elderly patients with diabetic peripheral vascular disease. Three RCTs assessed modified Huangqi Guizhi Wuwu Decoction and Tangmai Tongluo Decoction in the treatment of lower extremity vascular lesions, reporting improved blood flow to the affected limbs. In addition, Yiqi Tongluo Qingre cream demonstrated efficacy in improving microcirculation of the common carotid, popliteal, and dorsal foot arteries, while also enhancing lipid metabolism profiles. Four studies on proprietary Chinese patent medicines, including Naoxintong capsule, and Shexiang Baoxin pill, further supported the therapeutic potential of TCM in elderly populations. Moreover, four RCTs involving a total of 432 elderly patients evaluated extracts from notoginseng, and Salvia miltiorrhiza, Ginkgo biloba, confirming their clinical value in vascular health in this patient population (34).

Natural polysaccharides often complexed with proteins and polyphenols exert strong antioxidant effects (136). For instance, Pu’er tea polysaccharide (137) and Gastrodia elata polysaccharide (138) demonstrate potent antioxidant activity, while polysaccharides from lichii and Ampelopsis grossedentata, rich in uronic acid, show similar efficacy (139). Conversely, removing polyphenols and proteins from some sources, such as edible mushroom polysaccharides, markedly reduces their antioxidant potential (140).

At the molecular level, the Nrf2 pathway is central to oxidative stress defense. Nrf2, normally bound to Keap1 in the cytoplasm, undergoes rapid degradation under basal conditions (141). Polysaccharides can promote Nrf2 release, enabling nuclear translocation, activation of antioxidant response elements (ARE), and upregulation of downstream antioxidant enzymes, including phase II detoxifying enzymes. For example, Ostra rivularis polysaccharides enhance Nrf2/ARE gene expression (142), while Cyaeoleya polysaccharide (CPP) increases Nrf2 while decreasing Keap1 expression, with glycogen synthase kinase-3β (GSK3β) acting as an upstream regulator (143). In addition, PI3K/AKT phosphorylation can activate Nrf2 signaling and inhibit JNK and IRS1 expression, thereby suppressing oxidative damage (144). Angelica sinensis polysaccharides inhibit caspase-dependent apoptosis, while Schisandra chinensis polysaccharides modulate the MAPK pathway and mitochondrial apoptotic signaling by regulating p-JNK1, caspase-3, and cytochrome c while upregulating Bcl-2 (145).

Polysaccharides also regulate endogenous antioxidant enzymes such as superoxide dismutase (SOD), and glutathione peroxidase (GSH-Px), via the Nrf2/ARE axis (146, 147). Additionally, Lydium barbarum polysaccharides (LBP) scavenge free radicals and suppress oxidative stress, modulating lipid peroxidation and MDA formation while boosting SOD, and GSH-Px activity (148, 149). Astragalus polysaccharides likewise inhibit malondialdehyde production and increase antioxidant enzyme activity (150). Polysaccharides derived from Cordyceps sinensis demonstrate strong free radical scavenging abilities (DPPH, ABTS, and hydroxyl radical assays) (151, 152). Similarly, Angelica sinensis polysaccharides regulate mitochondrial function, increasing mitochondrial enzyme activity and ATP production (153). Other polysaccharides, including purslane-derived compounds, enhance SOD, and GSH-Px activity, reducing lipid peroxidation while regulating Nrf2-related gene expression (154, 155). Collectively, polysaccharides from multiple TCM herbs and food sources demonstrate potent antioxidant effects, modulating oxidative stress and reducing inflammation in diabetic complications (156).

Structural features such as monosaccharide composition, molecular weight, and sulfate content significantly influence antioxidant potency (151, 157). For instance, Laminaria japonica polysaccharide (LJP) demonstrates high antioxidant activity due to its sulfate content, and fucoidan from brown seaweed exhibits strong free radical scavenging activity even at high temperatures (157). Enzymatic or fermentation treatments can reduce molecular weight and enhance antioxidant activity, with mannuronic/guluronic acid ratios in alginate influencing activity as well (151, 152). Mushroom polysaccharides, including those from Lentinus edodes, Flammulina velutipes, Pleurotus ostreatus, and Grifola frondosa, also show potent free radical scavenging abilities (158, 159). Likewise, polysaccharides from Ganoderma lucidum, Hericium erinaceus, and Cordyceps sinensis activate antioxidant enzymes and modulate apoptosis-related proteins (160, 161). Probiotic-derived polysaccharides, such as those produced by Lactobacillus plantarum and Brevibacterium attiditis, exhibit strong antioxidant effects without cytotoxicity (162).

Marine- and animal-derived polysaccharides likewise demonstrate therapeutic potential. Misgurnus anguillicaudatus polysaccharide (MAP), chondroitin sulfate from sea cucumbers, and chitosan exhibit significant antioxidant activity by scavenging hydroxyl radicals and reducing oxidative damage (163, 164). Functional chemical modifications including sulfation, phosphorylation, carboxymethylation, and selenization further enhance water solubility, bioactivity, and antioxidant activity (136, 165). For example, sulfated polysaccharides from Auricularia auricula and Flammulina velutipes improve aging- and inflammation-related outcomes (136, 166), while phosphorylated pumpkin and ginseng polysaccharides enhance antioxidant enzyme capacity (167, 168). Similarly, carboxymethylated Sargassum fusiforme polysaccharides demonstrate improved free radical scavenging activity (168, 169).

## 4 Study quality and limitations

While the studies cited in this review provide valuable insights into the potential therapeutic effects of TCM in managing diabetic complications, it is important to note several limitations. A majority of the studies reviewed are preclinical or animal-based, which, although informative, may not fully reflect the complexities of human physiology. Animal models often fail to account for the variations seen in human metabolism, disease progression, and response to treatment.

Furthermore, there is a lack of large-scale human clinical trials to validate the findings from animal studies and preclinical research. Without comprehensive, well-designed human trials, it remains challenging to definitively establish the efficacy, safety, and long-term effects of these TCM formulations. The transition from animal studies to human populations is often complicated by differences in biological systems, making it difficult to directly translate animal-based results into practical human therapies.

While the potential of TCM in managing diabetic complications is promising, further research, particularly large-scale clinical trials, is necessary to confirm these effects and explore their applicability to human populations.

## 5 Conclusion

Extensive research has revealed the therapeutic potential and mechanistic basis of TCM in the management of T2DM and its associated complications. With the advancement of omics technologies, mass spectrometry, and network pharmacology, the bioactive components of TCM and their molecular targets are being increasingly elucidated. Nevertheless, further work is required to advance standardization, quality control, and clinical validation of TCM formulations, ensuring reproducible efficacy and clearly defined mechanisms of action.

Future investigations should prioritize the integration of modern medical techniques with TCM approaches, alongside exploration of synergistic strategies that combine TCM with Western medicine. Such collaborative frameworks could open new avenues for comprehensive treatment, ultimately benefiting a broader population of patients with T2DM. In parallel, TCM research should continue to deepen understanding of T2DM pathophysiology, while fostering greater international collaboration and knowledge exchange with the global medical community (170).

Overall, TCM represents considerable promise as both a therapeutic modality and a research tool, offering the potential to make a substantial and lasting contribution to human health and well-being worldwide.
