# Supplementary material for: Traditional Chinese medicine in diabetes management: a comprehensive review of mechanisms and therapeutic potential
Source: Front Endocrinol (Lausanne). 2026 Mar 3;17:1709404. doi: 10.3389/fendo.2026.1709404 (PMC12992034; doi:10.3389/fendo.2026.1709404)
Supplement: Supplementary file 1 [file Table1.docx]

**Supplementary Tables**

| Herb (Scientific Name) | Common / Pinyin Name |
| --- | --- |
| Rehmannia glutinosa Libosch | Di Huang |
| Ophiopogon japonicus | Mai Dong |
| Poria cocos | Fu Ling |
| Panax ginseng | Ren Shen |
| Astragalus membranaceus | Huang Qi |
| Glycyrrhiza uralensis Fisch. | Gan Cao |
| Dioscorea opposita | Shan Yao |
| Schisandra chinensis | Wu Weizi |
| Cornus officinalis | Shan Zhuyu |
| Alisma orientale | Ze Xie |
| Trichosanthes kirilowii | Gua Lou |
| Anemarrhena asphodeloides | Zhi Mu |
| Scrophularia ningpoensis | Xuan Shen |
| Pueraria lobata | Ge Gen |
| Lycium barbarum | Gou Qi |
| Paeonia lactiflora | Bai Shao |
| Angelica sinensis | Dang Gui |
| Coptis chinensis | Huang Lian |
| Salvia miltiorrhiza | Dan Shen |
| Codonopsis pilosula | Dang Shen |
| Atractylodes macrocephala | Bai Zhu |
| Atractylodes lancea | Cang Zhu |

**Supplementary Table 1.** Frequently Used Herbal Medicines (List 1)

| Herb (Scientific Name) | Common / Pinyin Name |
| --- | --- |
| Astragalus membranaceus | Huang Qi |
| Rehmannia glutinosa Libosch | Di Huang |
| Trichosanthes kirilowii Maxim | Gua Lou |
| Schisandra chinensis | Wu Weizi |
| Dioscorea opposita Thunb. | Shan Yao |
| Ophiopogon japonicus | Mai Dong |
| Panax ginseng | Ren Shen |
| Pueraria | Ge Gen |
| Poria cocos | Fu Ling |
| Anemarrhena asphodeloides Bge | Zhi Mu |
| Lycium barbarum L. | Gou Qi |
| Glycyrrhiza uralensis Fisch. | Gan Cao |
| Salvia miltiorrhiza | Dan Shen |
| Scrophularia ningpoensis Hemsl. | Xuan Shen |
| Cornus officinalis Sieb. et Zucc. | Shan Zhuyu |
| Alisma orientale | Ze Xie |
| Polygonatum sibiricum Red. | Huang Jing |
| Coptis chinensis Franch. | Huang Lian |
| Pseudostellaria heterophylla (Miq.) Pax et Hoffm. | Taizi Shen |
| Paeonia suffruticosa Andr. cortex, dried | Shao Yao |
| Polygonatum odoratum (Mill.) Druce | Yu Zhu |
| Lonicera japonica Thunb. | Jin Yinhua |
| Zea mays | Yu Mi Xu |
| Psidium guajava L. | Fan Shi Liu |

**Supplementary Table 1.** Frequently Used Herbal Medicines (List 2)

**Table 2.** Thematic synthesis of mechanistic evidence for TCM interventions in T2DM.

| Theme | Synthesis (pathways, examples, evidence) |
| --- | --- |
| 1. Insulin secretion / incretin response | Convergent reports describe improved beta-cell responsiveness and glucose-stimulated insulin release, with representative interventions including berberine-containing Coptis chinensis preparations [9; 10]. Evidence is mechanistically rich (cell/animal) but clinically heterogeneous. |
| 2. beta-cell preservation + intestinal carbohydrate handling | Frequently framed as reduced beta-cell stress/apoptosis together with lower postprandial glucose via alpha-glucosidase/alpha-amylase inhibition (e.g., SQC, puerarin, Morus alba leaf/Sang Ye) [7]. Evidence is strongest for biochemical and preclinical endpoints; clinical standardization remains limited. |
| 3. Insulin sensitivity and glucose uptake | Many interventions converge on AMPK activation and restoration of PI3K/Akt-GLUT4 signaling, aligning mechanistic observations across muscle, adipose, and liver. Preclinical replication is common; fewer well-powered RCTs use standardized preparations or direct measures of insulin sensitivity. |
| 4. Lipid metabolism and ectopic fat (obesity and MASLD/MAFLD) | Synthesized findings emphasize improved hepatic lipid handling (reduced lipogenesis, increased oxidation) and lower adipose inflammation, overlapping with AMPK/PPAR and bile-acid related pathways. Clinical relevance is high, but endpoints are often biomarker-based rather than imaging or long-term outcomes. |
| 5. Inflammation/oxidative stress and mitochondrial homeostasis | Across organs, effects repeatedly map to Nrf2 activation and suppression of NF-kB/NLRP3 and AGE-RAGE-related injury; polysaccharides and anti-inflammatory phytochemicals are common exemplars [147]. Mechanistic plausibility is strong, but human pathway engagement is rarely confirmed. |
| 6. Systems-level remodeling (metabolomics/network approaches) | Metabolomics studies of classical prescriptions suggest coordinated shifts in bile acids, branched-chain amino acids, and lipid intermediates consistent with multi-pathway engagement [24]. These data are hypothesis-generating and should be triangulated with clinical outcomes and mechanistic biomarkers. |

**Table 3.** Condensed synthesis of complication-focused clinical evidence and mechanistic convergence.

| Complication domain | Synthesis (approaches, mechanisms, evidence) |
| --- | --- |
| Nephropathy | Relatively largest clinical base: meta-analyses of RCTs evaluate multi-herb prescriptions/patent medicines, often as add-on therapy, with improvements mainly in renal injury surrogates (e.g., albuminuria/proteinuria) in some studies [34]. Mechanistic convergence: anti-inflammatory/antioxidant and anti-fibrotic pathways. Key limitations: heterogeneity, variable trial quality, and limited hard endpoints. |
| Retinopathy | Clinical evidence is limited (few studies in meta-analyses in older adults) with variable ophthalmic outcomes and follow-up; mechanistic work emphasizes microvascular protection, oxidative stress/inflammation control, and barrier stabilization. Key limitation: predominantly preclinical mechanistic support and inconsistent clinical endpoints. |
| Peripheral neuropathy | Clinical studies frequently report modest symptom and/or nerve conduction improvements, but endpoints and diagnostic criteria vary. Mechanistic convergence: reduced neuroinflammation/oxidative injury and improved microcirculation. Key limitation: need standardized, blinded assessments and durability data. |
| Cardiomyopathy / cardiovascular risk | Small clinical studies report improvements in functional surrogates when formulas are used adjunctively; mechanistic synthesis overlaps with endothelial protection and metabolic/redox remodeling. Key limitation: co-interventions and confounding; standardized cardiac endpoints and safety monitoring are needed. |
| Peripheral vascular disease | A small number of RCTs in older adults evaluate proprietary preparations as adjuncts; synthesized targets include microcirculatory and endothelial protection with anti-inflammatory effects. Key limitation: small samples and inconsistent reporting of randomization/blinding and product standardization. |
